# COVID-19 Peripheral Neuropathy: A Report of Three Cases

**DOI:** 10.7759/cureus.18132

**Published:** 2021-09-20

**Authors:** Keith B Diamond, Miriam D Weisberg, Mitchell K Ng, Orry Erez, David Edelstein

**Affiliations:** 1 Orthopaedic Surgery, Maimonides Medical Center, Brooklyn, USA

**Keywords:** covid-19, peripheral neuropathy, ulnar neuropathy, critical care polyneuropathy, ain neuropathy, lateral femoral cutaneous nerve, anterior interosseous nerve neuropathy, lfcn neuropathy, meralgia paresthetica

## Abstract

This study includes three patients with various peripheral neuropathies after contracting coronavirus disease 2019 (COVID-19) infection, treated both conservatively and surgically. While cases of neurological complications have been described, neuropathy associated with COVID-19 is under-reported in orthopaedic literature. These patients presented with ulnar neuropathy, critical care polyneuropathy (CCP) with anterior interosseous nerve (AIN) neuropathy, and lateral femoral cutaneous nerve (LFCN) neuropathy. COVID-19 infection may be associated with peripheral neuropathy in addition to various neurological sequelae. Orthopaedic surgeons should screen patients for recent infections and evaluate the severity of the illness to assess for risk of neurological sequelae of COVID-19 infection.

## Introduction

In December 2019, a rapidly transmitted unknown cause of viral pneumonia, soon named Severe Acute Respiratory Syndrome Coronavirus 2 (SARS-CoV-2) spread throughout China and to the rest of the world [1]. As the virus spread, the initial primary symptoms included fevers, myalgias, fatigue, and dry cough [2]. Further research revealed that many patients presented with neurological symptoms, rather than the typical respiratory symptoms, including headache, unsteady gait, cerebral infarction, cerebral hemorrhage, and other neurological diseases [3].

There are several reports of peripheral neuropathies associated with severe coronavirus disease 2019 (COVID-19), developing from compressive neuropathy, mixed central and peripheral nervous system disorders, symmetric polyneuropathy, and systemic effects from critical illness neuropathy [4-7]. Many patients develop severe disease, requiring ICU admission and extended hospitalization [8, 9]. With the advent of prone positioning for patients with COVID-19 acute respiratory distress syndrome (ARDS), intubated patients are put at risk for compressive neuropathies due to malposition of extremities [4,9-11]. In addition, neurologic symptomatology has been attributed to neuroinvasion, neurotropic characteristics of COVID-19, and neuroinflammatory events following infection [5-7,12].

With the myriad of neurological phenomena associated with COVID-19 infection, orthopaedic surgeons should have a diagnostic suspicion while evaluating patients presenting with new neurologic findings after recent illness of COVID-19. Patients should be screened for recent infection, including severity of illness and hospitalization, as part of a thorough history when assessing for risk of neurological sequelae following COVID-19 infection.

## Case presentation

Case 1. 

A 41-year-old previously healthy male presented to the emergency department in March 2020 with shortness of breath and fever, tested positive for COVID-19 on polymerase chain reaction (PCR) testing and was instructed to return home to quarantine. Six days later, the patient returned to the emergency department with acute hypoxic respiratory failure. The patient was intubated, then placed on extracorporeal membrane oxygenation (ECMO). His two-month ECMO course was complicated by bacteremia, shock, desynchrony from the vent, with intermittent use of paralysis and vasopressors. He was disconnected from ECMO, weaned from the vent, and discharged from the hospital in mid-June.

The patient was evaluated six months after discharge by a hand surgeon for complaints of left throbbing hand pain, inability to extend the small finger, and pain radiating to the elbow. On exam, the patient had five degrees of metacarpophalangeal (MCP) hyperextension with a flexion contracture of the small finger distal interphalangeal (DIP) and proximal interphalangeal (PIP), numbness of the small and ring fingers, with decreased sensation on the dorsal and palmar aspects of the ulnar hand. His motor exam revealed ulnar hand intrinsic strength to be 3/5. The cubital tunnel compression test was positive for exacerbating symptoms.

**Figure 1:**
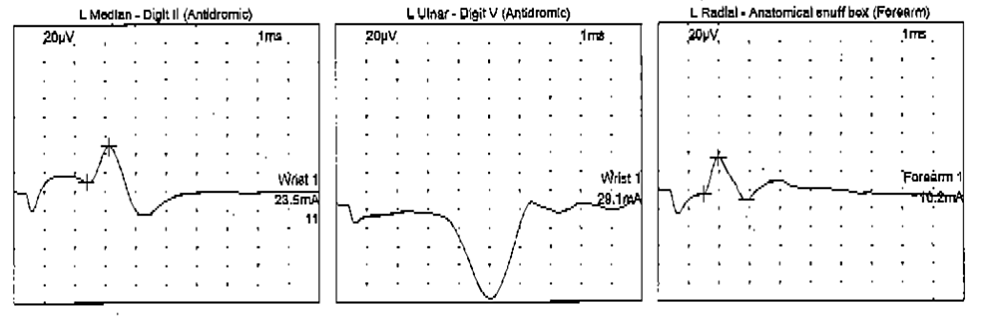
Sensory NCS revealed absent ulnar nerve response NCS: nerve conduction study

**Figure 2:**
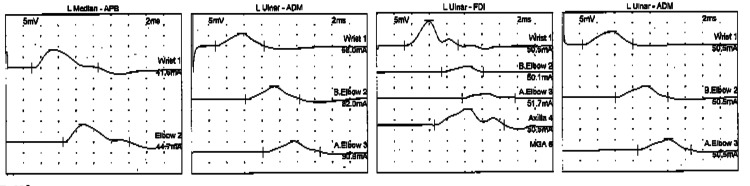
Motor NCS revealed significant slowing of the left ulnar nerve across the elbow when recorded from the abductor digiti minimi and first dorsal interosseous nerve NCS: nerve conduction study; APB: abductor pollicis brevis; ADM: abductor digiti minimi; FDI: first dorsal interosseous

He underwent imaging, nerve conduction studies (NCS), and electromyogram (EMG). Radiographs indicated clawing deformity of the small finger without evidence of fracture. Sensory NCS revealed slowing of the left ulnar nerve across the elbow, with absent ulnar nerve response (Figure 1). In addition, motor NCS identified mild slowing of the left ulnar nerve across the elbow (Figure 2). Furthermore, EMG showed mild chronic reinnervation changes of the left first dorsal interosseous nerve, with mild chronic reinnervation changes in the left flexor carpi ulnaris and flexor digitorum profundus.

The patient was subsequently diagnosed with ulnar neuropathy and treated with both cubital tunnel release and splinting of the small finger contracture. At six months post-op, the patient had improving numbness along dorsal and volar aspects of ulnar hand as well as 4+/5 intrinsic strength. He was placed in a finger cast for persistent flexion contracture of the small finger with minimal improvement.

Case 2.

A 73-year-old male with a past medical history of hypertension and hypercholesterolemia presented to the emergency department with severe COVID-19 infection in April 2020. He was admitted to the ICU and underwent intubation and tracheostomy during his hospital course, remaining on the ventilator for seven weeks. After being transferred and eventually discharged from rehab, he noticed severe weakness of his left hand, with the inability to close his fist. He denied pain, but he developed constant numbness of the left index and long fingers, with intermittent paresthesias of the entire left hand. He noted numbness of the right long finger as well. After resuming ambulation, he noticed a right foot drop without any associated back or leg pain.

He presented two months after discharge from rehab with complaints of bilateral hand pain. The patient complained of painful paresthesias of both upper extremities, worse with activities. On exam, he had a severe weakness (1-2/5) of left forearm pronation, wrist and finger flexion, with weakness of left finger abduction (3/5) and extension (4/5). There was also a weakness of left shoulder abduction, with mild diffuse weakness of the right hand. Bilateral thenar atrophy was present. The right lower extremity revealed weakness (4+/5) of right foot dorsiflexion. Sensation was diminished in the left median nerve distribution. Reflexes were hypoactive in the upper extremities and brisk at the knees and ankles. Plantar responses were downward going.

**Figure 3:**
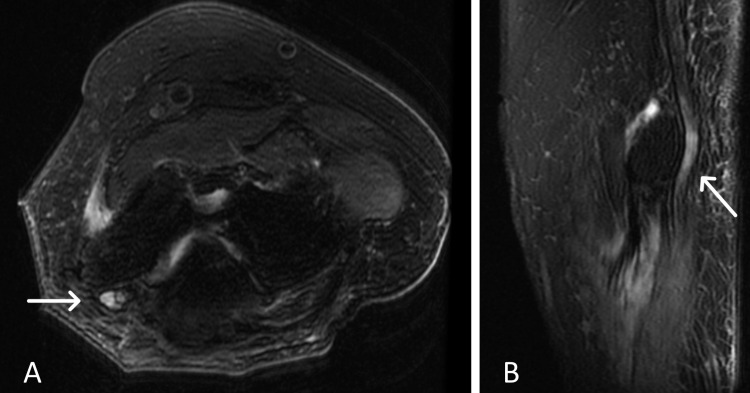
Axial (A) and Sagittal (B) MRI of the left elbow reveals thickening and increased signal intensity of the ulnar nerve proximally and at the level of the cubital tunnel, consistent with ulnar neuritis/cubital tunnel syndrome.

The patient was sent for imaging, EMG, and NCS for concern of general neuropathy, left median, and right peroneal entrapment neuropathies. MRI of the left elbow revealed thickening and increased signal intensity of the ulnar nerve proximally and at the level of the cubital tunnel, consistent with ulnar neuritis and cubital tunnel syndrome. Edematous were changes seen, involving the flexor muscle groups and pronator teres, as well as atrophy of the supinator muscle group, distal biceps, and brachialis, consistent with advanced denervation (Figure 3). EMG studies were suggestive of chronic severe generalized polyneuropathy. There was evidence of superimposed left median entrapment polyneuropathy localized proximally to the pronator muscle and non-localizable right deep peroneal entrapment neuropathies (Table 1).

**Table 1:** EMG studies suggestive of chronic severe generalized non-length dependent sensory and motor polyneuropathy, with no evidence of demyelinating polyneuropathy. There is electrophysiologic evidence of superimposed left median entrapment polyneuropathy localized proximally to the pronator muscle and non-localizable right deep peroneal entrapment neuropathies MUAP: motor unit action potential; Fasc: fasciculations; LA: latency of activation; Fib: fibrillations; PSW: polyspike wave; HF: high frequency; Amp: amplitude; PPP: polyphasic potential; N: normal

Needle EMG									
	Spontaneous					MUAP			Recruitment
	LA	Fib	PSW	Fasc	H.F.	Amp	Duration	PPP	Pattern
Left first dorsal interosseihand	N	None	None	None	None	N	Long	3+	Decreased
Left flexor pollicis longus	N	None	None	None	None				No Activity
Left flexor digitorum superficialis	N	None	None	None	None				No Activity
Left pronator teres	N	None	None	None	None				No Activity
Left extensor digitorum longus	N	None	None	None	None	N	Long	3+	Decreased
Left triceps	N	None	None	None	None	N	Long	3+	Decreased
Left biceps	N	None	None	None	None	N	Long	3+	Decreased
Right first dorsal interossei foot	N	None	None	None	None				No Activity
Right tibialis anterior	N	None	None	None	None	N	Long	3+	Decreased
Right tibialis posterior	N	None	None	None	None				Subopt Effort
Right gastrocnemius	N	None	None	None	None	N	Long	3+	Decreased
Right vast right vastus lateralis	N	None	None	None	None	N	Long	3+	Decreased

Given the imaging and electrodiagnostic studies, the clinical impression included critical care polyneuropathy (CCP), superimposed anterior interosseous nerve (AIN) neuropathy, and right peroneal entrapment neuropathy. The patient pursued conservative management, including physical therapy. At seven months follow-up, the patient reports persistent pain in the left hand, characterized by decreased strength and increased stiffness in the thumb, index, and long fingers. He reports resolution in right upper and lower extremity symptoms.

Case 3.

A 54-year-old male with a past medical history of obesity and asthma presented to the emergency department with severe COVID-19 infection in May 2020. The patient was intubated for six weeks, including prone position treatment during the hospital course. He reported feeling right lower extremity numbness after extubation in June, along with groin and lateral thigh pain. The patient suffered from constant right thigh pain and numbness for six months, described as diminishing. The pain and numbness slowly decreased in dermatomal distribution size over time.

On exam, the patient had a mildly antalgic gait. The patient had trochanteric tenderness, as well as pain with palpation along the lateral femoral cutaneous nerve distribution through the thigh. The range of motion was grossly intact with mild pain. Imaging was performed, including radiography and MRI. X-ray of the left hip showed evidence of arthritis, while MRI revealed gluteal tendinopathy, with soft tissue edema and bursitis.

The patient was diagnosed with lateral femoral cutaneous nerve (LFCN) neuropathy, secondary to prone positioning during hospital admission, along with bursitis and arthritis of the hip. The patient was treated conservatively, including meloxicam for pain control. At eight months follow-up, the patient reports decreased pain severity and distribution with noninvasive management.

## Discussion

As COVID-19 cases continue, more patients present with neurological sequelae following infection. This case series describes a variety of post-COVID-19 peripheral neuropathies, including ulnar neuropathy, CCP with superimposed AIN and peroneal neuropathies, and LFCN neuropathy. The causes of each patient’s presentation differ in pathophysiology. The pathophysiology behind the first patient is speculated by viral infiltration of the peripheral nervous system, while the second patient suffered from a generalized polyneuropathy from systemic metabolic derangements. The last patient presented with compressive neuropathy, highlighting the association between COVID-19 infection and prone positioning during ICU stays that may lead to the complication of peripheral nerve injury.

In each of our patients, there is an element of nerve compression that may contribute to their unique presentations. Our patient with LFCN neuropathy was treated with prone positioning during his prolonged hospital stay in the ICU. In their retrospective review of 83 patients treated with prone positioning for COVID-19 ARDS, Malik et al. identified 12 patients (14.5%) with subsequent peripheral neuropathy following extubation. Similar to the current case study, they had a single case of LFCN neuropathy in a patient, confirmed by EMG [13]. Amongst their 12 patients with peripheral neuropathy, five patients had ulnar neuropathy following prone positioning [13]. This is consistent with another retrospective review of 114 patients treated with prone positioning for COVID-19 ARDS that identified 30 cases of peripheral neuropathy, 12 of which included ulnar nerve injury [14]. Furthermore, Sayegh et al. describe successful conservative management of three patients presenting with ulnar neuropathy following prone treatment for ARDS, consisting of steroid taper and steroid injections [4]. While our patient with ulnar neuropathy was not treated with prone positioning, the possibility of compression upon the ulnar nerve for an extended period while intubated cannot be ruled out. Patients who are intubated and sedated cannot express the early symptoms of neurapraxia, thus the final manifestations of peripheral nerve injury are identified after eventual extubation and removal of sedation. Long-term effects have not yet been described [13,14].

It is possible that ulnar neuropathy is a product of viral infiltration of the peripheral nervous system [4]. There are several reports of COVID-19 infections of the peripheral nervous system, manifesting as symmetrical lower motor neuron polyneuropathy, mixed central and peripheral nervous system disorder, and Guillain-Barré Syndrome [1,5,6,15]. In their systematic review, Ellul et al. describe 19 cases of Guillain-Barré Syndrome, characterized by symmetrical limb weakness, areflexia, sensory disturbances, beginning at a median of seven days following respiratory or systemic COVID-19 illness [1]. COVID-19 has displayed various degrees of neurotropism, explaining its association with neurologic symptoms. The virus exhibits a spike protein (S), which binds angiotensin-converting enzyme 2 (ACE-2), which is present in olfactory epithelium [1,16]. Although the mode of neuroinvasion is not well established, it is proposed that peripheral nerve terminals of the olfactory and vagus nerves allow for viral entry into the CNS [12]. While there are limited studies describing isolated peripheral nerve involvement, this may be one of the first case reports to describe an isolated peripheral neuropathy following COVID-19 infection. Given the hypothesis that the patient’s ulnar neuropathy is a product of viral inflammation and compression, it is plausible that surgical treatment provided incomplete resolution of hand function because the pathogenesis of the underlying condition is intrinsic to the nerve, rather than solely as a compressive force.

It is important to suspect CCP when evaluating a patient that was intubated. CCP is a well-established polyneuropathy that presents in patients who undergo mechanical ventilation and prolonged ICU stays [17]. While the pathophysiology of CCP is not well established, one theory includes systemic inflammation and overproduction of cytokines, nitric oxide, and oxygen radicals, causing hypoxic and anaerobic conditions, leading to axonal degeneration due to decreased microcirculation [17,18]. Another theory describes chronic inflammation, causing changes in vascular permeability, leading to vasogenic edema [19,20]. CCP presents with muscle weakness, typically symmetrical, with sensory loss and areflexia, with failure to wean from ventilation in the ICU after sepsis [17,18,20]. As illustrated in the current study, EMG will show axonal loss without demyelinating features, while NCS will show the decreased amplitude of sensory nerve action potentials [17]. The unique presentation of superimposed AIN syndrome and peroneal entrapment upon CCP in our patient can be explained by the edematous changes in the flexor muscle groups and pronator teres described on MRI. Our patient may have acquired CCP during his prolonged ICU stay and developed isolated peripheral neuropathies as a byproduct of the compressive nature of peripheral edema [20]. Treatment of CCP should include reduction of dose and duration of neuromuscular blocking agents and corticosteroids, rehabilitation programs, careful extremity positioning, and antioxidant therapies [18].

When evaluating patients with new-onset peripheral neuropathy, orthopaedic surgeons should screen for recent COVID-19 infection, the severity of illness, history of intubation, and use of prone positioning treatment. COVID-19 related neuropathy should be included in an orthopaedist’s differential diagnosis, as the combination of early identification with non-invasive treatment can allow for more invasive procedures to be forgone. Conservative management is recommended for the treatment of peripheral neuropathy secondary to prone positioning as well as CCP. Further research must be performed regarding the pathophysiology of direct viral inoculation of the peripheral nervous system and subsequent management of the neurologic sequelae.

## Conclusions

This case series describes a variety of unique presentations of peripheral neuropathies after severe COVID-19 infection. Patients presented with ulnar neuropathy, critical illness polyneuropathy with superimposed anterior interosseous nerve peroneal nerve neuropathies, as well as lateral femoral cutaneous nerve neuropathy. With the high prevalence of COVID-19 infection worldwide, we suggest orthopaedic surgeons screen for COVID-19 infection history while evaluating patients with new-onset neuropathy.
